# Partial Purification and Characterization of Bioactive Peptides from Cooked New Zealand Green-Lipped Mussel (*Perna canaliculus)* Protein Hydrolyzates

**DOI:** 10.3390/foods9070879

**Published:** 2020-07-04

**Authors:** Ramya Jayaprakash, Conrad O. Perera

**Affiliations:** School of Chemical Sciences, The University of Auckland, Auckland 1010, New Zealand; rjay196@aucklanduni.ac.nz

**Keywords:** green-lipped mussel protein, enzyme hydrolysis, bioactive peptides ACE inhibitors, In-silico analysis

## Abstract

Proteins from fresh New Zealand green-lipped mussels were hydrolyzed for 240 min using pepsin and alcalase. The extent of the hydrolysis, antioxidant, antimicrobial, and angiotensin-converting enzyme (ACE) inhibitory activities of each protein hydrolysate were investigated. Peptides obtained from pepsin hydrolysis after 30 min, named GPH, exhibited the highest antioxidant and ACE inhibitory activity, but no antimicrobial activity. Purification of the GPH using gel-filtration chromatography revealed that the protein fraction (GPH-IV*) containing peptides with a molecular weight (MW) below 5 kDa had the strongest antioxidant and ACE inhibitory activities. Further purification was done using reverse-phase HPLC (RP-HPLC) and the only major peak obtained (GPH-IV*-P2) had the highest antioxidant and ACE inhibitory activity. From this fraction, several bioactive peptides with an MW ≈ 5 kDa were identified using LC-MS and in silico analyses. This research highlights that green-lipped mussel protein hydrolysates could be used as a good source of bioactive peptides with potential therapeutic applications.

## 1. Introduction

Green-lipped mussel (*Perna canaliculus)* is a bivalve mollusk endemic to New Zealand. Historically, it is a well-known staple food of the Māori population living in the coastal regions. Researchers have observed that the coastal Māori were less prone to osteoarthritis than the inland Māori population, which inspired them to investigate the nutritional values of *P. canaliculus* [1].

Several studies related to the production of bioactive peptides from the marine mussel proteins revealed that enzymatic hydrolysis was one of the cheapest and most reliable methods of producing bioactive peptides [2,3,4,5]. Moreover, the enzymatic digestion of the mussel proteins is well-known to produce potential bioactive peptides with 2–30 amino acids, enhancing their absorption into the intestine. This could favor the direct entry of the peptides into the circulatory system and exert the necessary biological responses at the target site [6]. However, to our knowledge, no such information is available in the literature on New Zealand green-lipped mussels (GLM).

In general, some of the most common proteolytic enzymes used for protein hydrolysis are alcalase, pepsin, flavorzyme, Neutrase, papain, and Protamex [7,8,9,10]. The production of bioactive peptides is greatly influenced by several factors, such as the pH, degree of hydrolysis (DH), enzymes used, enzyme/substrate ratio, temperature, hydrolysis time, and the solvents used [9,11].

New Zealand produces nearly 100,000 tonnes of GLM a year, worth close to $350 million. Although many dietary supplements and nutritional products are manufactured from the export rejects of mussels, there are also opportunities for manufacturing high-value natural therapeutics to extend the application range of GLM.

The key objectives of this study were to evaluate an efficient method of production of bioactive peptides from NZ green-lipped mussels and to evaluate their biological properties, such as antioxidant activity, angiotensin-converting enzyme (ACE) inhibitory activity, and antimicrobial properties. Based on the results, the best bioactive protein hydrolysate was selected for further purification using gel-filtration chromatography and RP-HPLC analysis. The fraction showing the strongest antioxidant and ACE inhibitory activities were identified using LC-MS/MS and their partial characterization was done using in silico methods.

## 2. Materials and Methods

### 2.1. Materials

Live New Zealand green-lipped mussels (*Perna Canaliculus*) were purchased from a local supermarket in Auckland (36.8485° S and 174.7633° E), New Zealand. They were brought to the laboratory and stored in a freezer at −20 °C before further treatment. They were used within one week of purchase. Pepsin from porcine gastric mucosa (3200–4500 u/mg), Alcalase^®^ CLEA (≥5 u/g), and Angiotensin-I converting enzyme (ACE) from rabbit lung (≥2 u/mg) were purchased from Sigma Aldrich (St Louis, MO, USA). Bovine serum albumin (fraction V low endotoxin) was purchased from Gibco-BRL (Life Technologies, Needham, MA, USA).

### 2.2. Preparation of Green-Lipped Mussel Proteins for Enzymatic Hydrolysis

The flesh was separated manually from the shells and byssus threads, and a sample of 50 g of mussel tissue was washed thoroughly with water to remove salt and other impurities. It was boiled for 10 min to inactivate the endogenous proteases [9] and was homogenized at 1500 rpm for 5 min into a fine paste using a homogenizer (Kinematica GmbH, Steinhofhalde 20–22, CH-6005 Luzern, Switzerland). The protein content was measured using the modified Bradford assay technique [12] using 8 M urea containing 2% β-mercaptoethanol to resolubilize the protein, and bovine serum albumin (BSA) was used to prepare the standard curve.

### 2.3. Enzymatic Processing of Mussel Proteins

Two protease enzymes, pepsin and alcalase, were used for the hydrolysis process. A sample of 20 g of homogenized mussel prepared as above was taken in each of four 500 mL beakers. MilliQ water was added to each of the four beakers in the ratio of 1:10 (*w*/*v*) mussel to water. For the hydrolysis by pepsin, the pH of the mussel homogenates with added water in two of the four beakers was adjusted to 2.5 by adding 1 M HCl, and for alcalase, the pH was adjusted to 8.5 using 1 M NaOH in the other two beakers. The reaction was initiated by adding either pepsin or alcalase at an enzyme to protein (substrate) ratio of 1:10 (*w*/*w*). One of the two beakers with added pepsin was incubated at 37 °C and the other was incubated at 60 °C. The same procedure was followed with the other two beakers with the added alcalase.

The pH of the two enzymatic reactions was monitored using a pH meter (perpHecT LogR meter, model 320, Wasson Victor, Ltd, Beverly, MA, USA) and remained constant via the addition of either 1 M HCl or 1M NaOH throughout the experiment. Enzymatic hydrolysis was carried out for four hours and samples (10 mL) were withdrawn every 30 min (30, 60, 90, 120, 150, 180, 210, 240 min) and heated to 100 °C for 10 min to inactivate the enzymes. The pH of the heated hydrolysates was adjusted to 7 after cooling to room temperature. The samples were centrifuged at 12,000× *g* for 10 min using a Sorvall Lynx 4000 (Thermoscientific Centrifuge Inc., Franklin, MA, USA). The supernatant was collected, freeze-dried, and stored at −20 °C until further use.

### 2.4. Determination of the Percentage Degree of Hydrolysis (DH(%))

The degree of hydrolysis was measured using OPA (O-phthalaldehyde) according to the method of Nielsen et al. [13]. Spectrophotometric analysis was carried out with the help of an Enspire multimode reader (Perkin Elmer Inc., Waltham, MA, USA) at 340 nm. Samples of 100 mg of freeze-dried pepsin or alcalase hydrolysates were weighed into volumetric flasks and made up to 10 mL with MilliQ water to obtain a 10 mg/mL concentration.

An aliquot of 10 µL of each sample and standard was pipetted into a 96-well Optiplate (Perkin Elmer Inc., Waltham, MA. USA) in triplicate. An aliquot of 100 µL of freshly prepared OPA reagent was pipetted into each well. It was incubated at room temperature for 5 min and absorbance readings were taken at 340 nm. MilliQ water was used as the blank. The mean value of the absorbance readings obtained from three assays was used for the calculations. The DH was calculated using *H* and *H_tot_* values, as given by Nielsen et al. [13]:

$$\mathrm{DH}=\frac{H}{H_{tot}\;}\times 100\%,$$

where $H=\frac{\left(\mathrm{Leucine}-{\mathrm{NH}}_{2}-\beta \right)}{\alpha }$ meqv/g protein, *H_tot_* is 7.77 meqv/g of meat, α is 1.0, and β is 0.4, as given by Nielsen et al. [13]. The determination of *H* depends on the leucine-NH_2_ value which can be expressed as:

$$\mathrm{Leucine}-{\mathrm{NH}}_{2}=\frac{\left(A\left(\mathrm{sample}\right)-A\left(\mathrm{blank}\right)\right)}{\left(A\left(\mathrm{standard}\right)-A\left(\mathrm{blank}\right)\right)}\times 0.762\left(\frac{\mathrm{meqv}}{L}\right)\times 0.1\times \frac{100}{\left(X\times P\right)},$$

where leucine-NH_2_ is in meqv leucine-NH_2_/g of protein; *X* is the amount of the sample (in g); *P* is the percent of protein present in the sample, 0.1 L is the volume of the sample, and 0.762 meqv/L is the equivalent weight of leucine.

### 2.5. Biological Activities of Protein Hydrolyzates

The biological activities of mussel protein hydrolysates were studied by determining their antioxidant, ACE inhibitory, and antimicrobial activities.

#### 2.5.1. DPPH (1,1-Diphenyl-2-Picrylhydrazyl) Radical Scavenging Activity

The DPPH activity was measured according to the method of Bougatef et al. [14] with slight modifications. Spectrophotometric analysis was carried out with the help of an Enspire multimode plate reader at 517 nm. An aliquot of 10 µL each of the samples (10 mg/mL concentration) and the standard was pipetted into a 96-well microtiter plate in triplicates. An aliquot of 200 µL DPPH reagent was pipetted into each well. It was incubated at room temperature for 5 min in the dark and absorbance readings were taken at 517 nm. MilliQ water was used as the blank. The mean value of the obtained absorbance readings was used for the calculations. The DPPH radical scavenging activity percentage was calculated using the following formula:

$$\mathrm{DPPH}\;\mathrm{radical}\;\mathrm{scavenging}\;\%=\frac{A\left(\mathrm{blank}\right)-A\left(\mathrm{sample}/\mathrm{standard}\right)}{A\left(\mathrm{blank}\right)}\times 100,$$

where *A*(blank) is the absorbance readings of blank and *A*(sample/standard) is the absorbance readings of enzymatic hydrolysates or Trolox standards.

#### 2.5.2. ABTS (2, 2′-Azino-Bis-Ethylbenzthiazoline-6-Sulfonic Acid) Radical Scavenging Activity

The ABTS radical scavenging activity was measured according to the method of Yang et al. [15]. Spectrophotometric analysis was carried out with the help of an Enspire multimode reader at 734 nm. An aliquot of 10 µL each of the samples and the standard was pipetted into a 96-well microtiter plate in triplicate. An aliquot of 200 µL of working solution of ABTS reagent was pipetted into each well. It was incubated at room temperature for 10 min in the dark and absorbance readings were taken at 734 nm. MilliQ water was used as the blank. The mean value of the obtained absorbance readings was used for the calculations. The ABTS radical scavenging activity percentage was calculated using the following formula:

$$\mathrm{DPPH}\;\mathrm{radical}\;\mathrm{scavenging}\;\%=\frac{A\left(\mathrm{blank}\right)-A\left(\mathrm{sample}/\mathrm{standard}\right)}{A\left(\mathrm{blank}\right)}\times 100,$$

where *A*(blank) is the absorbance readings of the blank and *A*(sample/standard) is the absorbance readings of enzymatic hydrolysates or Trolox standards.

#### 2.5.3. Angiotensin-Converting Enzyme (ACE) Inhibitory Activity Assay

The in vitro ACE inhibitory activity assay was performed using a microplate fluorometric assay according to the method of Schwager et al. [16] with the following modifications. The assay was based on the hydrolysis of hippuryl-L-hystidyl-L-leucine (HHL) using ACE to form hippuric acid and histidyl-leucine (HL). The reaction was stopped via the addition of NaOH, the HL produced was reacted with OPA, and the fluorescence was detected at an excitation wavelength of 385 nm and an emission of 485 nm.

The assay buffer (pH 8.3) comprised potassium di-hydrogen phosphate (0.1 M), di-sodium hydrogen phosphate (0.1 M), and zinc chloride (10.0 mM).

Briefly, 20 μL of 100 mU/mL ACE solution was incubated with 50 μL of the assay buffer containing 5 mM HHL and 10 μL of various concentrations (7.8 nM to 62.5 nM) of a known ACE inhibitor, Captopril (Fluka Chemie AG, Buchs, Switzerland), or the freeze-dried hydrolyzed or unhydrolyzed mussel protein samples (10 mg/mL). The reaction was conducted at 37 °C for 30 min and then stopped by adding 50 μL of 1.0 M aqueous NaOH. An aliquot of 50 μL of 20 mg/mL OPA reagent was reacted with the HL produced for 10 min, and the fluorescence intensity was measured at a 385 nm excitation and a 485 nm emission.

The ACE inhibitory activity percentage was calculated using the following equation:

$$\mathrm{ACE}\;\mathrm{inhibitory}\;\mathrm{activity}\;\%=\frac{\mathrm{Fluorescence}\;\mathrm{intensity}\;\mathrm{of}\;\left(\left(\mathrm{sample}/\mathrm{standard}\right)-\;\mathrm{blank}\right)\;}{\mathrm{Fluorescence}\;\mathrm{intensity}\;\mathrm{of}\;\left(100\%\;\mathrm{activity}\;-\mathrm{blank}\right)\;}\times 100.$$

#### 2.5.4. Antimicrobial Activity Assay

The antimicrobial assay was performed in the Faculty of Medical and Health Science (FMHS), Grafton Campus, The University of Auckland. Minimal inhibitory concentration (MIC), minimal bactericidal concentration (MBC), and minimal fungicidal concentration (MFC) tests were used for measuring the lowest concentration of mussel proteins required to inhibit growth or kill all bacteria or fungi in a suspension, respectively. The MIC test was performed against 1 × 10^6^ CFU (colony forming units)/mL of *Escherichia coli* (ATCC 25922), *Bacillus cereus* (ATCC 10702), *Staphylococcus aureus* (ATCC 6538), and *Candida albicans* in a 96-well microplate, according to the method of Eloff [17]. The MIC is defined as the lowest concentration of an antimicrobial that will inhibit the visible growth of a microorganism after overnight incubation, and MBC or MFC is defined as the lowest concentration of an antimicrobial that will prevent the growth of an organism after subculture onto antibiotic-free media [18].

### 2.6. Gel Filtration Chromatography of Bioactive Peptides

The mussel protein hydrolysates showing the highest antioxidant activity and ACE inhibitory activity were further purified using gel filtration chromatography and RP-HPLC analysis. The proteins of the green-lipped mussel hydrolysates (GPHs) were separated using a Sephadex G-25 gel filtration column (2.5 × 30 cm) (Merck KgaA, Darmstadt, Germany) which was previously prepared and equilibrated with MilliQ water. The column was eluted with MilliQ water at a flow rate of 1 mL/min, 5 mL fractions were collected, and the absorbance was measured at 215 nm. A graph was plotted for the absorbance of protein fractions vs. the fraction number. Protein fractions were pooled into eight fractions (GPH-I to GPH-VIII) and lyophilized. The antioxidant and ACE inhibitory assays were carried out on the freeze-dried protein fractions.

### 2.7. SDS Polyacrylamide Gel Electrophoresis (SDS-PAGE)

SDS-PAGE was performed using 4–15% mini-PROTEAN^®^ TGX™ precast protean gels with a 10-well comb at 50 µL/well purchased from Bio-Rad (Bio-Rad laboratories Pty., North Harbour, Auckland, New Zealand). SDS-PAGE was used for the determination of the molecular weight of the unhydrolyzed green-lipped mussel proteins, 30 min pepsin enzymatic hydrolysates, and protein fractions obtained using gel filtration chromatography. A sample of 10 mg of each the unhydrolyzed green-lipped mussel proteins, 30 min pepsin hydrolysates, and protein fraction obtained using gel-filtration (GPH-IV) was weighed out from the corresponding freeze-dried powders and dissolved in 1 mL of MilliQ water. An aliquot of 50 µL of the prepared sample was mixed with 50 µL of an SDS reducing sample buffer. Samples and the standards prepared with SDS reducing sample buffer were incubated at 95 °C for 5 min and cooled down to room temperature (20 °C) before adding 20 µL into each well.

### 2.8. RP-HPLC Analysis of Bioactive Protein Fractions

The highest bioactive protein fraction obtained from the gel-filtration was purified using RP-HPLC (Agilent 1100 HPLC, Agilent Ltd, Santa Rosa, CA, USA) on a Zorbax SB C-18 column (4.6 × 250 mm, 5 μm; Agilent, Santa Clara, CA, USA). Initially, the Zorbax column was conditioned for 60 min using eluent A (0.05% trifluoroacetic acid). Then, following the injection of 40 µL of a bioactive protein fraction, a linear gradient of eluent B (100% acetonitrile) (0–60% for 30 min) at a flow rate of 1 mL/min was used for elution of the peaks, which were detected at 215 nm. Each fraction was separated and collected every 1 min (from the retention time of 5 min to 20 min). The collected peptide fractions were lyophilized and the bioactivity assays were carried on the freeze-dried peptide fractions.

### 2.9. Identification of Bioactive Peptides Using LC-MS/MS

Identification of the RP-HPLC purified bioactive peptide fraction using LC-MS/MS was carried out by Auckland Science Analytical Services, The University of Auckland. The highest bioactive peptide fraction, at the concentration of 5 mg/mL, was diluted 50-fold with 0.1% formic acid. An aliquot of a 10 µL sample was injected and the peptides captured were desalted on a 0.3 × 5 mm PepMap cartridge (LC Packings, Dionex Corporation, Sunnyvale, CA, USA) before being separated on a 0.3 × 100 mm Zorbax 300SB-C18 column. For the analysis of the bioactive fraction (GPH-IV*-P2) from the RP-HPLC purification, the HPLC gradient between buffer A (0.1% formic acid in water) and buffer B (0.1% formic acid in acetonitrile) was formed at 6 μL/min as follows: 10% B for the first 3 min, increasing to 35% B up to 24 min, increasing to 97% B up to 26 min, held at 97% until 29 min, reduced to 10% B at 30.5 min, and held there until 35 min. The LC effluent was directed into the ion spray source of a QSTAR XL hybrid Quadrupole-Time-of-Flight mass spectrometer (Applied Biosystems, Foster City, CA, USA) scanning from 330–1600 m/z. The top three most abundant multiple-charged ions were selected for MS/MS analysis (100–1600 m/z). The mass spectrometer and HPLC system were under the control of the Analyst QS 2.0 software package (Applied Biosystems, Foster City, CA, USA).

### 2.10. In Silico Analysis of Bioactive Peptides Isolated from New Zealand Green-Lipped Mussels

A de novo transcriptome assembly was performed on mussel gill tissue using Trinity (trinityrnaseq.github.io). TransDecoder (transdecoder.github.io) was subsequently used to identify the longest possible open reading frames for genes in the transcriptome. These genes were then translated into protein-coding sequences for the database. The MS/MS data were searched against this database (22,714 entries in total) using Protein Pilot version 5.0 (Sciex) (Framingham, MA, USA) with the following parameters: search effort, thorough; cys alkylation, none; digestion, none; instrument type, QSTAR ESI.

BLAST (Basic Local Alignment Search Tool) (National Centre for Biotechnology Information, US National Library of Medicine, Bethesda, MD, USA) was used for determining the similarities between the peptides identified and the available protein sequences of all mollusks. In addition to that, the ExPASy tool (expasy.org) was used to compute the various parameters, such as the pI (isoelectric point), GRAVY (grand average of hydropathicity), amino acid and atomic composition, aliphatic index, instability index, and estimated shelf-life of each peptide sequence.

### 2.11. Statistical Analysis

All assays were carried out in triplicate and the values presented as mean ± standard deviation. Statistical analyses were performed using GraphPad Prism version 7.00 for Windows (GraphPad Software, La Jolla, CA, USA). Duncan′s test at *p* < 0.05 was conducted using SPSS 25.0 206 (IBM Inc., Armonk, NY, USA) to evaluate the statistical significance of the differences between means.

## 3. Results and Discussion

The green-lipped mussel protein was separately hydrolyzed using two commercial proteolytic enzymes, namely, alcalase and pepsin. Alcalase is an alkaline protease that can potentially generate bioactive peptides, as well as produce protein hydrolysates with higher nutritional and functional characteristics [19]. Pepsin is an acidic protease that can be successfully employed for the enrichment of bioactive peptides and the production of peptides, mostly with a short chain length, but also di- and tripeptides [20].

### 3.1. Determination of the Degree of Hydrolysis (DH(%))

**Figure 1:**
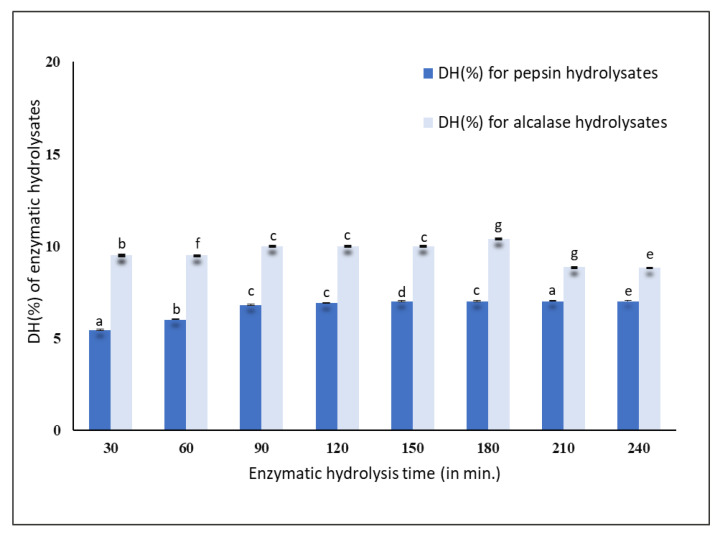
Determination of the degree of hydrolysis (DH (%)) of enzymatic hydrolysates using the OPA (O-phthalaldehyde) method. Values and error bars indicate the means and standard deviations, respectively, of triplicate analyses for each of the pepsin and alcalase hydrolysates. Values with different superscripts (a, b, c, …) indicate significant differences between the sample means at *p* < 0.05.

The results obtained for the pepsin hydrolysis indicated that within 30 min, the mussel protein was hydrolyzed to an extent of 5.45 ± 0.02%, after which it gradually increased to 6.90 ± 0.03% at 120 min. Later, it reached a steady phase with a DH (%) of 7.00 ± 0.02% after 240 min of hydrolysis, as shown in Figure 1. Alcalase hydrolyzed the mussel proteins to an extent of 8–10% up to 240 min. After 30 min of hydrolysis, the DH (%) was found to be 9.50 ± 0.05% and reached a steady phase with a DH (%) of 10.00 ± 0.02% up to 180 min and decreased to 8.00 ± 0.07% at 240 min, as shown in Figure 1. Therefore, it was considered that within 30 min, most of the proteins were hydrolyzed by the pepsin and alcalase enzymes.

The results obtained were similar to those obtained by Dai et al. [4] with *Mytilus edulis* (blue mussel). They found that hydrolysis took place at a rapid rate within 30 min using six different proteases, namely, alcalase, papain, Protamex, flavorzyme, Neutrase, and trypsin. They observed that there was a maximum cleavage of proteins within 30 min which tended to gradually decrease with an increase in the hydrolyzing time. Using yellow stripe trevally, Klompong et al. [2] found that DH (%) gradually increased when hydrolyzed for 5 to 20 min and reached a steady phase within 20 min, after which no further hydrolysis took place. Zhu et al. [19] observed that there was a high rate of hydrolysis within 1 h and increasing the hydrolysis time did not produce any significant changes in DH (%) after 3 h of hydrolysis of wheat germ protein. Therefore, the 30 min hydrolysis of New Zealand green-lipped mussels was considered adequate for producing protein hydrolysates.

### 3.2. DPPH (1,1-Diphenyl-2-Picrylhydrazyl) Radical Scavenging Activity

**Figure 2:**
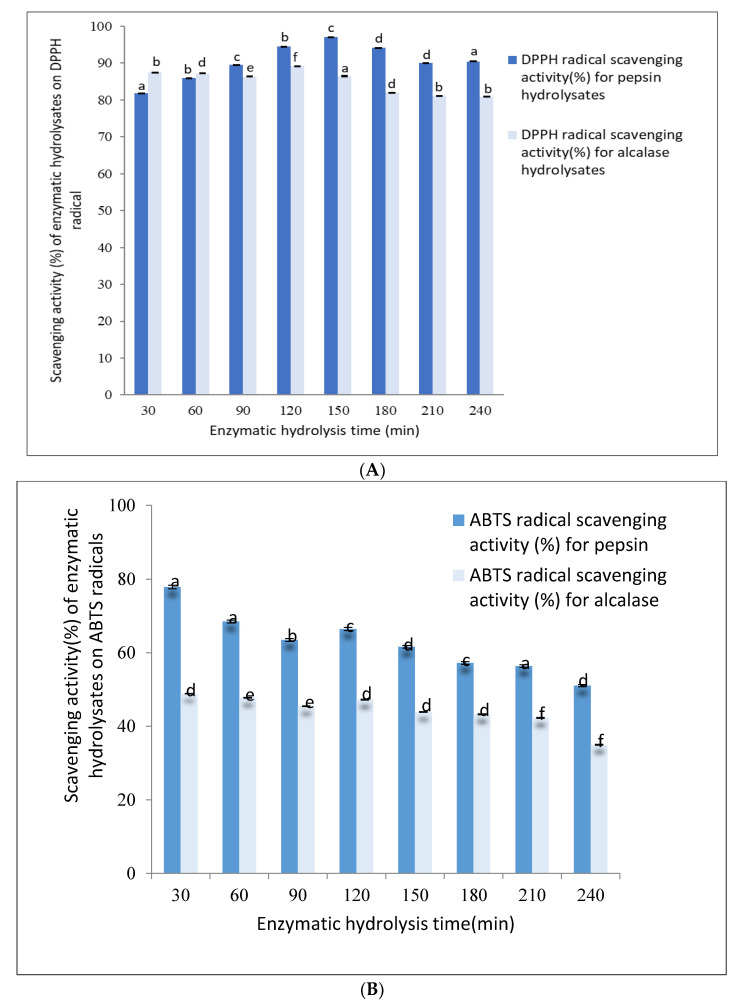
Antioxidant activities of pepsin and alcalase hydrolysates on (**A**) DPPH (1,1-diphenyl-2-picrylhydrazyl) and (**B**) ABTS (2,2′-azino-bis-ethylbenzthiazoline-6-sulfonic acid) radicals. Values with error bars indicate the means and standard deviations, respectively, of triplicate analyses. Values with different superscripts (a, b, c, …) indicate significant differences between the sample means at *p* < 0.05.

The DPPH radical scavenging activity of pepsin hydrolysates ranged from 82–97% up to 240 min of hydrolysis, as shown in Figure 2A. The DPPH radical scavenging activity of 30 min pepsin hydrolysates was found to be 82.00 ± 0.08% and gradually increased to 97.00 ± 0.01% after 150 min of hydrolysis. Further hydrolysis up to 240 min decreased the DPPH to 90.00 ± 0.06%.

The DPPH radical scavenging activity (%) of alcalase hydrolysates ranged from 87–89% when hydrolyzed up to 240 min, as shown in Figure 2A. The DPPH activity after 30 min of hydrolysis was found to be 87.00 ± 0.01%, remained constant until about 120 min, and gradually decreased to 80.00 ± 0.01% after 240 min.

The results obtained indicated that the antioxidant activities of the protein hydrolysates were influenced by the hydrolysis time and the enzyme used. The antioxidant activity of the pepsin and alcalase protein hydrolysates increased slightly until 120 min and 150 min of digestion, respectively, but decreased gradually when further hydrolyzed up to 240 min. This decrease in antioxidant activity may have been due to the loss of biological activity by the further hydrolysis of the bioactive peptides. The DPPH activities of enzyme hydrolysates produced using pepsin and alcalase were similar in that they were able to scavenge DPPH radicals effectively at around 82–87%.

### 3.3. ABTS (2,2′-Azino-Bis-Ethylbenzthiazoline-6-Sulfonic Acid) Radical Scavenging Activity

The pepsin hydrolysate obtained after 30 min of digestion was able to scavenge ABTS radicals up to 77.00 ± 0.04%, exhibiting the highest antioxidant ability, which gradually decreased to about 50% after 240 min of hydrolysis.

Similarly, the alcalase hydrolysate obtained after 30 min had the highest ABTS radical scavenging activity of 50.00 ± 0.01%. As with the pepsin hydrolysates, the ABTS radical scavenging activity of alcalase hydrolysates decreased gradually to 38.00 ± 0.01% after 240 min of hydrolysis, as shown in Figure 2B.

Wang et al. [5] obtained similar results during the hydrolysis of *M. edulis* (blue mussel) using alcalase. They found that the antioxidant properties of blue mussel protein hydrolysates were greatly influenced by the time and enzyme used for hydrolysis. The radical scavenging effects of the peptides were lost during prolonged hydrolysis times due to the high degree of hydrolysis [7]. Working with the mussel variety *Perna viridis* (Asian green mussel), Ismail and Hasni [7] found that bioactive peptides with a high MW (>6 kDa) had stronger radical scavenging activity than low-MW peptides, and a high degree of hydrolysis resulted in the loss of antioxidant properties of the peptides. Working with yellow stripe trevally proteins using alcalase and flavorzyme, Klompong et al. [2] found that the antioxidant activity decreased with an increase in DH.

Based on the current study, it can be clearly stated that a hydrolysis time of 30 min was sufficient to generate potential antioxidant peptides from *P. canaliculus* (New Zealand green-lipped mussel) proteins using pepsin or alcalase.

### 3.4. ACE Inhibitory Activity Assay

**Figure 3:**
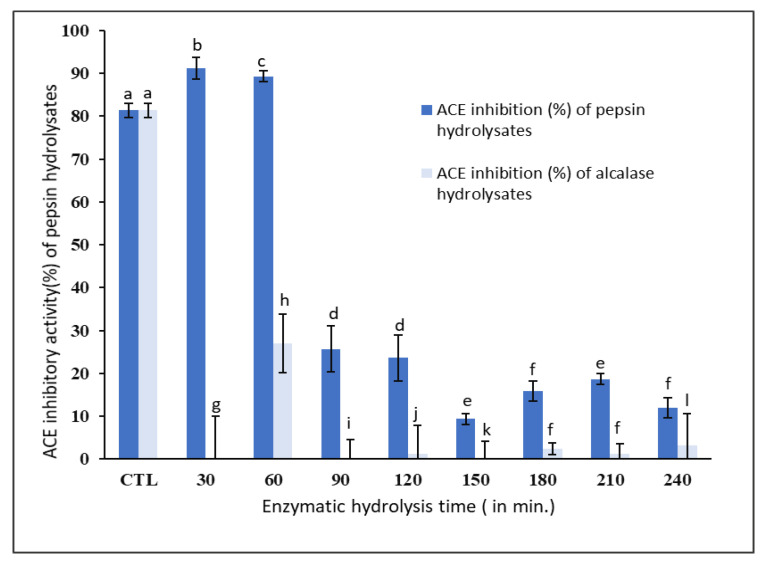
Percentage angiotensin-converting enzyme (ACE) inhibitory activity of pepsin and alcalase hydrolysates. Values with different superscripts (a, b, c, ….) indicate significant differences between sample means at *p* < 0.05. The control (CTL) was captopril.

The ACE inhibitory activities of pepsin and alcalase hydrolysates in comparison to captopril, a standard inhibitor of ACE activity, are shown in Figure 3. As expected, the ACE inhibition increased with an increase in the concentration of captopril (Figure S1).

The ACE inhibitory activity of unhydrolyzed *P. canaliculus* proteins (control) was found to be 81.0 ± 1.7%. However, the ACE inhibitory activity increased to 91.0 ± 2.5% after 30 min of hydrolysis using pepsin and it did not significantly (*p* < 0.05) change after 60 min of hydrolysis (Figure 3). On the other hand, the ACE inhibitory activity of alcalase hydrolysates was much lower (5.0 ± 0.2%) compared to the pepsin hydrolysates and was also much lower than the unhydrolyzed protein isolate (Figure 3).

Lee et al. [3] compared the ACE inhibitory activity of tuna frame protein hydrolysates using six proteases, namely, pepsin, alcalase, papain, Neutrase, trypsin, and α-chymotrypsin. They found that tuna protein hydrolyzed by pepsin for 8 h had the highest ACE inhibitory activity of 88.2%, whereas alcalase hydrolysates showed an ACE inhibitory activity of only 48%. Dai et al. [4] found that the enzyme hydrolysates of *M. edulis* (blue mussel), which had lower DH of 5%, exhibited the strongest ACE inhibitory activity. They also found that the ACE inhibitory activity of enzyme hydrolysates decreased at a rapid rate with increasing DH. Jamadar et al. [21] showed that the ACE inhibitory activity of peanut protein hydrolysates using alcalase reached an optimum ACE inhibition of around 10% DH and no significant rise in ACE inhibition was observed with an increase in DH. Tsai et al. [22] hydrolyzed hot-water-extracted hard clam protein using the protease Protamex and found that the peptides having a molecular weight range of 300–350 Da had the highest ACE inhibitory activity.

The current study clearly showed that 30 min pepsin hydrolysates, with a DH of 5.45 ± 0.02%, had the strongest ACE inhibitory activity of 91.0 ± 2.5%, as well as antioxidant activity of 82.0 ± 0.1% against DPPH radicals and 77.0 ± 0.04% against ABTS radicals. Since the ACE inhibitory activity of 30 min alcalase hydrolysates was very low (5.0 ± 0.2%), the 30 min pepsin hydrolysates were considered to have the most potential bioactivity. Therefore, the peptides produced via 30 min pepsin hydrolysis were purified further using GPC and RP-HPLC for further characterization with LC-MS/MS.

### 3.5. Antimicrobial Activity Assay

The unhydrolyzed *P. canaliculus* (New Zealand green-lipped mussel) protein and their hydrolysates after 30 min of pepsin and alcalase digestion did not show any antimicrobial properties. Attempts to evaluate the turbidity of the microorganisms on day 2 failed as no difference was observed in the formation of turbidity in the test wells and the negative control wells. To confirm the results, spotting was done by plating the liquid suspension from the test wells and the negative controls. The tests were concluded as negative since the microbial colonies were formed in all sections containing different concentrations of mussel protein and the protein hydrolysates.

This result might have been due to the absence of antimicrobial peptides in New Zealand green-lipped mussels or the presence of antimicrobial peptides in an inactive form in the precursor mussel protein. Another reason could have been due to the presence of other metabolites conjugated with the protein structure or the requirement of further solvent extraction other than water for the release of antimicrobial peptides. However, Annamalai et al. [23] and Grienke et al. [1] identified one antimicrobial peptide (9.7 kDa) from the *P. viridis* species of mussel that had antibacterial activity against almost all pathogens compared to water extracts. They used solvents such as ethanol and methanol to isolate the proteins.

In a study using different bivalves from different countries, Rozanska et al. [24] observed that New Zealand green-lipped mussel protein did not possess any antimicrobial properties, which is similar to what was found in the present study. They also used confirmed the analysis of the peptides using HPLC-MS/MS for other mollusks, which showed positive results for antimicrobial activity. Sugesh and Mayavu [25] analyzed the antimicrobial activity of tissues extracted from edible salt-water clams *Meretrix casta* and *Meretrix meretrix* with ethanol, methanol, and acetic acid. They found that methanolic extracts of these two species showed high antimicrobial activities against all pathogens tested.

In the current study, only the water extracts of New Zealand green-lipped mussel (*P. canaliculus*) protein and protein hydrolysates were used, and they did not possess any antimicrobial activity. However, since most of the research on evaluating the antimicrobial activity of mollusks were carried out on the hemolymph, plasma, or specific tissues using organic solvents [26,27,28,29], such solvents and tissues from *P. canaliculus* could be used to extract protein, which would enhance the chance of identifying antimicrobial peptides. Crustaceans possess an open circulatory system, where nutrients, oxygen, hormones, and cells are distributed in the hemolymph. They lack an adaptive immune system and rely exclusively on their innate immune mechanisms that include both cellular and humoral responses [30].

### 3.6. Purification of Bioactive Peptides Using Gel Permeation Chromatography

**Figure 4:**
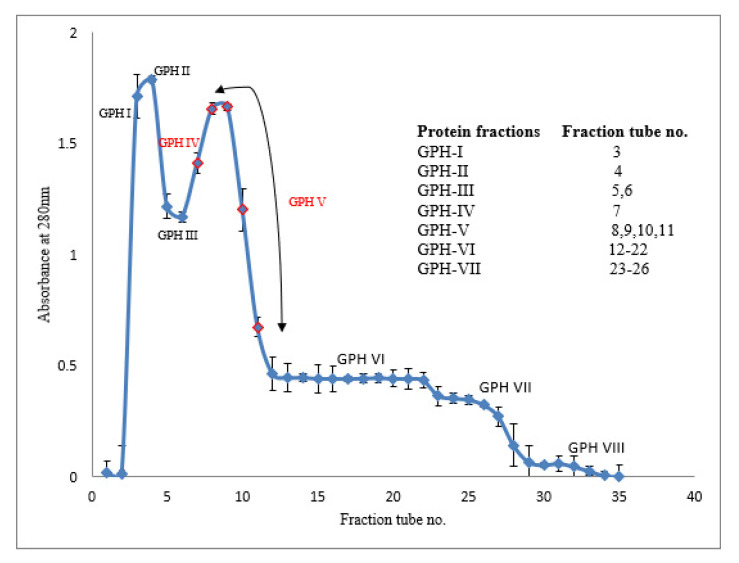
Gel permeation chromatography (GPC) of 30 min pepsin hydrolysates on a Sephadex G-25 column. Values and error bars indicate the means and standard deviations, respectively, of triplicate analyses for each protein fraction. Values highlighted in red indicate the highest bioactive protein fractions (GPH-IV and GPH-V).

Because the antioxidant activity and ACE inhibitory activity of alcalase-hydrolyzed proteins were much lower than those of pepsin-hydrolyzed proteins after 30 min, further purification of the peptides by gel permeation chromatography (GPC) was undertaken only on the latter hydrolysates. The GPC elusion pattern of the green-lipped mussel hydrolysates (GPHs) obtained after 30 min of pepsin digestion is shown in Figure 4. The collected protein fractions were pooled into eight sub-fractions (GPH-I to GPH-VIII) and their bioactivities were evaluated.

Antioxidant activities of the eluted protein fractions (GPH-I to GPH-VIII) were measured using DPPH and ABTS radical scavenging assays. Among the eight protein fractions, GPH-IV and GPH-V, exhibited the highest DPPH radical scavenging activities of 74.0 ± 0.1% and 70.0 ± 0.03%, respectively. These two protein fractions were also able to scavenge ABTS radicals stronger than the other protein fractions. GPH-IV and GPH-V showed the highest ABTS radical scavenging activities of 85.0 ± 0.01% and 75.0 ± 0.02% respectively.

The ACE inhibitory activity of all protein fractions GPH-I to GPH-VIII was above 60% but the fraction GPH-IV exhibited the highest ACE inhibitory activity of 94.0 ± 0.00%.

The fractions GPH-IV and GPH-V were the intermediate peptide fractions (with several low-MW proteins <1000 kDa) constituting the second major peak in the gel-permeation chromatogram (Figure 4). Therefore, these fractions were pooled together as GPH-IV* and further purified.

### 3.7. Characterization of the GPH-IV* Fraction from the Gel Filtration Using SDS-PAGE

**Figure 5:**
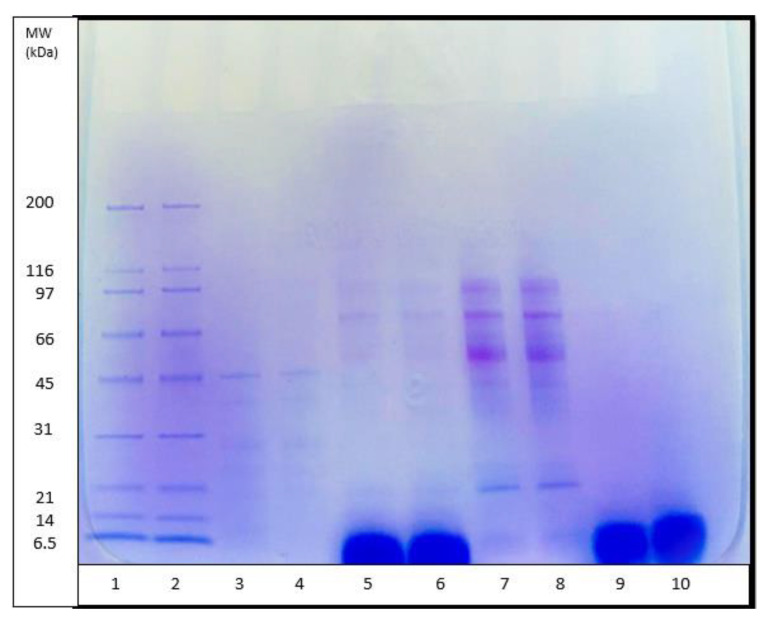
SDS polyacrylamide gel electrophoresis (SDS-PAGE) pattern of *P. canaliculus* proteins: lanes 1 and 2—broad-range standard protein marker (MW range of 200–6.5 kDa), lanes 3 and 4—unhydrolyzed green-lipped mussel protein, lanes 5 and 6—30 min pepsin hydrolysates, lanes 7 and 8—30 min alcalase hydrolysates, lanes 9 and 10—GPH-IV (protein fraction with the highest bioactivity).

The molecular weight (MW) distribution of unhydrolyzed *P. canaliculus* proteins, proteins hydrolyzed for 30 min using pepsin and alcalase, and GPH-IV* (the most potent bioactive protein fraction from the GPC) were determined using SDS-PAGE analysis (Figure 5). Thirty minutes of hydrolysis using pepsin produced large amounts of peptides in the range of 5 kDa, and small amounts of peptides in the range 97–14 kDa; meanwhile, alcalase produced large amounts of peptides in the high molecular weight range and very low amounts of short peptide fragments around 5 kDa.

The DH (%) using pepsin (Figure 1) was significantly lower than that using alcalase. However, the SDS-PAGE pattern of pepsin hydrolysates showed that most of the peptides were in the low-MW range, whereas those from alcalase had a relatively low level of low-MW peptides and a higher amount of medium-range MW peptides. The antioxidant properties of pepsin hydrolysates were significantly greater than those from alcalase hydrolysates (Figure 2A,B). It was found that pepsin hydrolysates also had stronger ACE inhibitory activity than alcalase hydrolysates. This could be attributed to the release of lower-MW peptides by pepsin hydrolysis than by alcalase hydrolysis. Salampessy et al. [31] analyzed the ACE inhibitory activity of yellow-stripe trevally proteins and showed that peptides with MW < 5 kDa exhibited the strongest ACE inhibitory activity. In another study, Raghavan and Kristinsson [32] observed that a low-MW (<10 kDa) peptide fraction of tilapia protein hydrolysates showed higher ACE inhibitory activity than high-MW (>30 kDa) peptides. The SDS-PAGE results for GPH-IV* indicated the presence of low-MW (<5 kDa) peptides in abundance.

### 3.8. RP-HPLC Analysis of Bioactive Protein Fraction (GPH-IV*)

**Figure 6:**
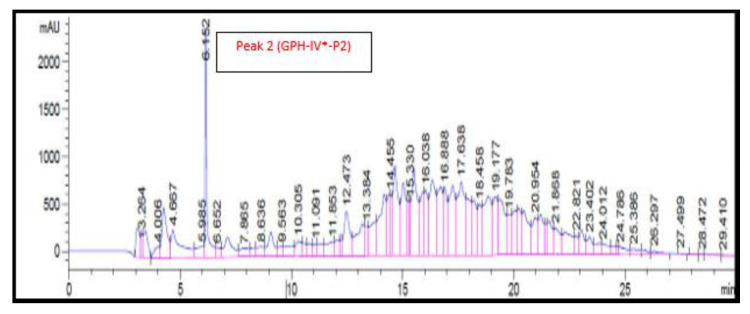
RP-HPLC chromatogram of the bioactive protein fraction (GPH-IV*). The peptides from the bioactive fraction (GPH-IV*) were separated using RP-HPLC on a Zorbax C-18 (4.6 × 250 mm, 5 μm) column. The separation was performed with a linear gradient (from 0–60% for 30 min) at a flow rate of 1 mL/min and monitored at 215 nm. The experiment was repeated 10 times and 15 peaks were collected every 1 min (from 5 to 20 min). The bioactivities were evaluated using a 5 mg/mL concentration of each peak. Peak 2 (GPH-IV*-P2) had the highest bioactivity.

The RP-HPLC chromatogram of the GPC-purified protein fraction (GPH-IV*) is shown in Figure 6. There was only one large peak, denoted by GPH-IV*-P2, after 6 min of elution; no other distinct peaks were identified in the RP-HPLC chromatogram. This might have been due to the presence of peptides with almost similar molecular weights and the difficulty in separating them in a big column [31].

The antioxidant activities of GPH-IV*-P2 were measured and were found to have a DPPH activity of 17 ± 0.01% and an ABTS activity of 16 ± 0.002%. The antioxidant activities of other peaks were significantly lower.

The ACE inhibitory activity of GPH-IV*-P2 was the highest at 96 ± 0.52%. There was an increase in the ACE inhibitory activity after the RP-HPLC purification, whereas the antioxidant property decreased, which might have been due to the removal of antioxidant peptides from the hydrolysates. Sarmadi and Ismail [33] showed that in some cases, there might be a loss of antioxidant properties of peptides due to the loss of sugar, peptides, or amino acids in the gel permeation process. However, the ACE inhibitory activity of protein fraction from gel filtration (GPH-IV*) with a value of 94 ± 0.0013% was increased to 96 ± 0.52% after the RP-HPLC purification (GPH-IV*-P2). This might have been due to the enhancement of the short-chain peptides in the purified fraction GPH-IV*-P2. Di Bernardini et al. [6] found that bioactive peptides with 2–20 amino acids possessed the strongest ACE inhibitory activities. The biological activities of the protein hydrolysates and purified peptide fractions using GPC and RP-HPLC are given in Table 1. In short, GPC and RP-HPLC purification resulted in the production of peptides with much higher biological activities. As seen from Figure 5 (lanes 9 and 10), they also consisted of low-MW peptide fractions.

### 3.9. Identification of the Bioactive Peptide Fraction Using LC-MS/MS

**Figure 7:**
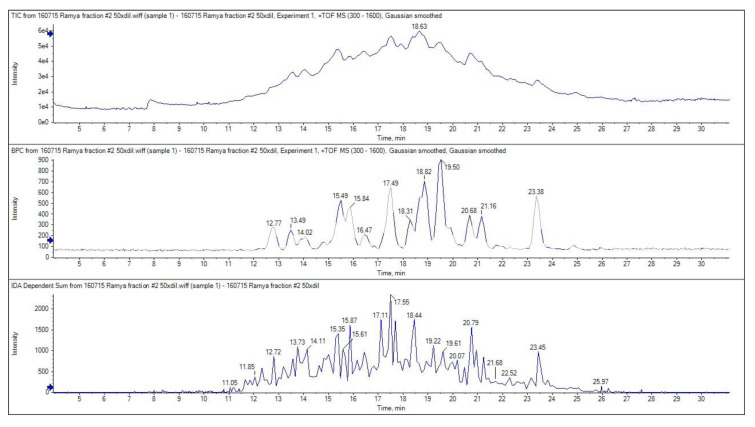
MS/MS spectra of bioactive peptides present in GPH-IV*-P2: (**top**) total ion chromatogram, (**middle**) base peak chromatogram, and (**bottom**) total MS/MS spectrum.

The ion chromatogram of GPH-IV*-P2 is shown in Figure 7. The top three most abundant multiple-charged ions were selected for MS/MS analysis with 100–1600 m/z and scanned through a 300–1600 m/z range.

The top panel in Figure 7 shows the total ion chromatogram, the middle panel shows the base peak chromatogram, and the bottom panel shows the MS/MS spectrum. The LC-MS results revealed that the highest bioactivity of peak 2 (GPH-IV*-P2) from the RP-HPLC purification was due to the presence of several lower-MW peptides. The peptides that showed high signal intensities in the MS analysis are listed in Table 2, along with their theoretical MW and observed MW obtained from the MS/MS spectrum. Wang et al. [5] found that peptides with 2–10 amino acids usually exerted the strongest bioactive properties due to their increased possibility to interact with free radicals. This may be attributed to the greater bioactive potentials of these peptides. Moreover, the smaller the peptide length, the higher the possibility for it to enter the biological cells and exert its bioactive properties.

The similarity matches and the sequence comparison with existing known sequences of other mollusk peptides for the peptide sequences obtained were identified using the standard protein BLAST (Basic Local Alignment Search Tool) software in NCBI (National Center for Biotechnology Information) [34] and are listed in Table 2. It was observed that the peptide sequences obtained were similar to those of the sarcoplasmic protein of Pacific oyster (*Crassostrea gigas*), the actin of California sea hare (*Aplysia californica*), and the collagen-like protein from Korean mussels (*Mytilus coruscus*). Based on this rapid analysis using BLAST, it could be seen that mollusks serve as a potential source of bioactive peptides.

The peptides showing the highest intensities and highest contributions in the MS/MS spectrum are also listed in Table 2. Moreover, the pIs (isoelectric points), GRAVY (grand average of hydropathicity) values, amino acid compositions, and the amino acids present at the N-terminal are also presented in Table 2. In addition, the atomic composition, instability index, estimated half-life, extinction coefficients, and aliphatic index of each peptide sequence could also be determined using the ExPASy tool from the SIB (Swiss Institute of Bioinformatics) resource portal [35]. This enables the understanding of the functional properties and chemical nature of each peptide sequence, which could be potentially used for the synthesis and further evaluation of their biological properties.

The bioactivity of each peptide could be predicted with the help of the BIOPEP database [36]. The pI value of each peptide sequence could be potentially used for the separation of peptides using ion exchange chromatography, determining the solubility of peptides, and for the characterization of particular peptides based on their net charge (neutral, acidic, or basic).

## 4. Conclusions

As reported by previous researchers, the production of bioactive peptides is greatly influenced by factors such as the pH, degree of hydrolysis (DH), enzymes used, enzyme/substrate ratio, temperature, hydrolysis time, and solvents used [9,11]. In this experiment, pepsin and alcalase were used to hydrolyze New Zealand green-lipped mussel proteins.

Thirty minutes of hydrolysis using pepsin produced large amounts of small-molecular-weight peptides with MW ≈ 5 kDa and small amounts of peptides in the range 97–14 kDa; meanwhile, alcalase produced large amounts of peptides in the high-molecular-weight range and very low amounts of short peptide fragments with MW ≈ 5 kDa. The antioxidant properties of pepsin hydrolysates were significantly greater than those from alcalase hydrolysates (Figure 2A,B). Thirty-minute pepsin hydrolysates also had a stronger ACE inhibitory activity than those produced using alcalase. Therefore, 30 min pepsin hydrolysates were purified further using RP-HPLC and the single peak obtained was subjected to LC-MS/MS analysis.

From the results obtained, it was observed that the peptides present in the bioactive fraction obtained through RP-HPLC purification had numerous short peptides with MW < 5 kDa. Furthermore, in silico analysis (using the ExPASy tool) of the amino acid composition for these peptides suggested that there was an abundance of hydrophobic amino acids, such as Gly, Val, Lys, Ile, and Ala, in the peptide sequences compared to the presence of other polar and charged amino acids, which might have contributed to the high bioactivity observed [37]. The pepsin treatment of green-lipped mussels serves as a cheap and effective means of producing these bioactive peptides rather than synthetic adjuncts. Bioactive peptides with the strongest radical scavenging activity and ACE inhibitory activity would provide a promising solution for the control of cardiovascular diseases.

In vivo studies related to these peptides exerting relevant bioactivity should be studied further since they might be susceptible to hydrolytic degradations or modifications by intracellular peptidases [38]. The application of bioinformatics provides a promising solution for less time-consuming and more efficient separation of peptides based on their functional characteristics. Therefore, classic methods of isolating bioactive peptides from food proteins combined with an in silico method of identifying them proved to be successful in the production of therapeutic agents (the antioxidant and ACE inhibitors) from green-lipped mussels.

Furthermore, research work related to the purification of these bioactive peptides and in vivo physiological relevance of these bioactive peptides should be carried out to confirm their biological properties and to develop potential natural therapeutic drugs. Green-lipped mussels, which are an abundant resource found in New Zealand waters, could be an inexpensive raw material for producing bioactive peptides, in contrast to the synthetic peptides.

## Figures and Tables

**Table 1:** Summary of the bioactivities of different hydrolysates and after each purification step. N/D: Not Determined.

Bioactivities	30 min Hydrolysates		GPC GPH-IV (%)	RP-HPLC GPH-IV*(P2) (%)
	Pepsin (GPH) (%)	Alcalase (%)		
**DH**	5.45 ± 0.02	9.5 ± 0.05	-	-
**DPPH Radical Scavenging Activity**	82 ± 0.08	87 ± 0.01	74 ± 0.13	17 ± 0.01
**ABTS Radical Scavenging Activity**	77 ± 0.04	80 ± 0.01	85 ± 0.03	16 ± 0.02
**ACE Inhibition**	91 ± 2.54	5 ± 0.21	94 ± 0.01	96 ± 0.52
**Antimicrobial Activity**	Nil	Nil	N/D	N/D

**Table 2:** Peptide sequence and other properties. AA: Amino Acid, MW: Molecular Weight, BLAST: Basic Local Alignment Search Tool, GRAVY: Grand Average of Hydropathicity.

AA Sequence	Theoretical MW	Observed MW	Best BLAST Hit	pI	GRAVY Value	Amino Acid Composition	Amino Acid Present at the N-Terminal
VDINRDGVVSE	1201.59	1201.58	Sarcoplasmic calcium-binding protein (*Crassostrea gigas*)	4.03	−0.2363	Asp: 18%; Val: 27.3%; Arg: 9.1%; Asn: 9.1%; Ser: 9.1%; Ile: 9.1%	Val-
AGDDAPRAVF	1017.49	1017.48	Predicted: actin, cytoplasmic (*Aplysia californica*)	4.21	−0.11	Ala: 30%; Asp: 20%; Arg, Gly, Val, Phe, Pro: 10%	Ala-
AVDINRDGVVSE	1272.63	1272.62	Sarcoplasmic calcium-binding protein (*Crassostrea gigas*)	4.03	−0.06	Val: 25%; Asp: 16.7%; Ala, Arg, Asn, Ser: 8.3%;	Ala-
FKIVDVND	948.49	948.50	Sarcoplasmic calcium-binding protein (*Crassostrea gigas*)	4.21	0.1625	Val, Asp: 25%; Phe, Lys, Ile, Asn: 12.5%	Phe-
DSGDGVTHTVPIYEG	1545.69	1545.69	Predicted: actin, cytoplasmic (*Aplysia californica*)	4.02	−0.473	Gly: 20%; Asp, Val, Thr: 13.3%, Pro, Ser, Tyr, His, Ile: 6.7%	Asp-
FKIVDVNDDKL	1304.70	1304.69	Sarcoplasmic calcium-binding protein (*Crassostrea gigas*)	4.43	−0.209	Asp: 27.3%; Lys, Val: 18.2%; Ile, Asn, Phe: 9.1%	Phe-
AIGVGPEVNQSQL	1310.68	1310.69	collagen-like protein-2 (*Mytilus coruscus*)	4	0.1	Gly, Gln, Val: 15.4%; Glu, Ile, Pro, Ser, Leu, Ala, Asn: 7.7%	Ala-
VGMGQKDSYVGDEAQSKRGILT	2338.16	2338.13	Predicted: actin, cytoplasmic (*Aplysia californica*)	6.09	−0.663	Gly: 18.2%; Asp, Gln, Lys, Ser, Val: 9.1%; Tyr, Met, Ile, Leu, Glu, Ala, Arg: 4.5%	Val-
KIKIIAPPERKYSVW	1827.08	1827.05	Predicted: actin, cytoplasmic (*Aplysia californica*)	10	−0.4266	Lys, Ile: 20%; Pro: 13.3%; Trp, Tyr, Val, Glu, Ala, Arg: 6.7%	Lys-
YKAFTHEDEKL	1379.67	1379.66	Sarcoplasmic calcium-binding protein (*Crassostrea gigas*)	5.45	−1.3727	Glu, Lys: 18.2%; Thr, Tyr, Phe, His, Asp, Ala, Leu: 9.1%	Tyr-

## Supplementary Materials

The following are available online at https://www.mdpi.com/2304-8158/9/7/879/s1, Figure S1: Standard curve of ACE inhibitory activity using Captopril.
