# Supplementary figures and images for: Partial Purification and Characterization of Bioactive Peptides from Cooked New Zealand Green-Lipped Mussel (Perna canaliculus) Protein Hydrolyzates
Source: Foods. 2020 Jul 4;9(7):879. doi: 10.3390/foods9070879 (PMC7404561; doi:10.3390/foods9070879)

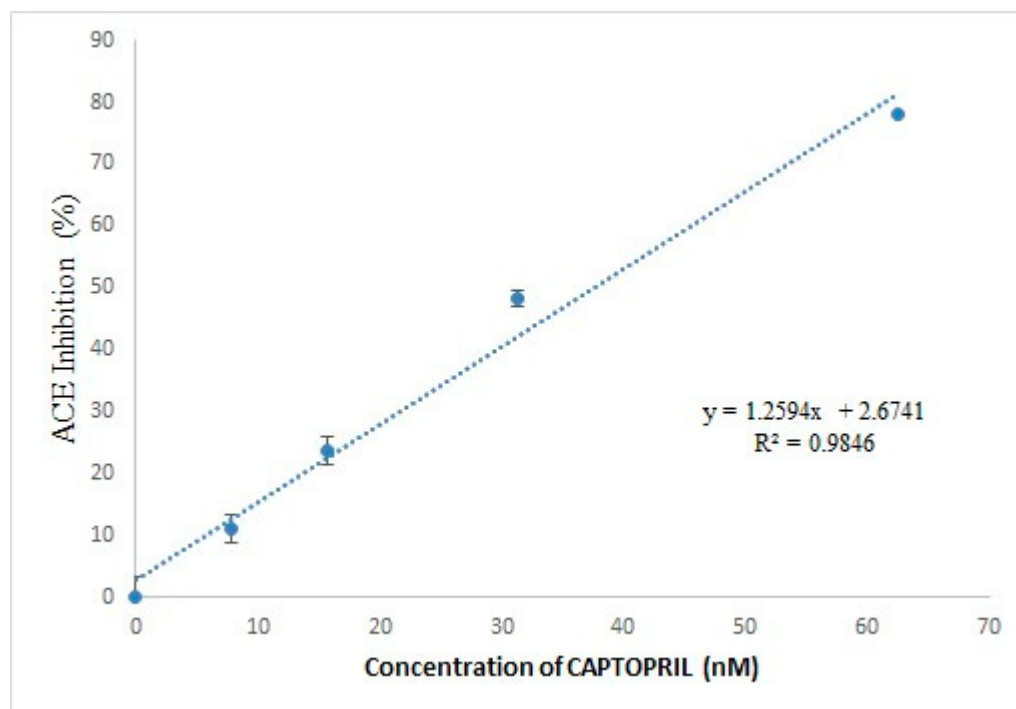

Figure S1. Standard curve of ACE inhibition using Captopril.

Supplement: Supplementary file 1 [file foods-09-00879-s001.pdf]
